# Current State and Prospects of Telemedicine in Morocco: Analysis of Challenges, Initiatives, and Regulatory Framework

**DOI:** 10.7759/cureus.50963

**Published:** 2023-12-22

**Authors:** Manar Jallal, Zineb Serhier, Hind Berrami, Mohamed Bennani Othmani

**Affiliations:** 1 Medical Informatics Department, 20 August Hospital, Casablanca, MAR

**Keywords:** telediagnostics, telehealth platforms, remote healthcare, teleconsultation, disparities, shortage, regulation, morocco, telemedicine, e-health

## Abstract

The Moroccan healthcare system is grappling with a pronounced lack of resources, particularly in terms of human personnel. Presently, Morocco has 28,892 doctors, which equates to a ratio of around 7.8 doctors per 10,000 inhabitants, whereas the WHO recommends a minimum of 23 doctors per 10,000 inhabitants. More than half of these doctors work along the Casablanca-Rabat axis, underscoring a significant disparity between urban and rural areas. In addition, about 270 rural municipalities find themselves in a state of critical medical isolation, denoting their location more than an hour away from a hospital facility. Among these municipalities, 160 are classified as priority, encompassing roughly two million inhabitants. Hence, the Moroccan healthcare system is confronted with several challenges in ensuring equitable access to quality services and curbing the escalating costs. Telemedicine holds the potential to address these twin needs by optimizing the utilization of existing human and material resources through telecommunications. In fact, telemedicine enables a reimagining of the healthcare landscape, promoting a territorial rebalancing in favor of regions with lower medical density. In this context, Morocco has established a regulatory framework outlining the rules for telemedicine practice. Numerous initiatives have emerged, particularly within the public sector, such as the National Telemedicine Initiative launched in October 2018, with the aim of covering 80% of medical deserts in Morocco by 2025. Nevertheless, despite the concerted efforts, there remain challenges to overcome in order to make strides and achieve the objectives set forth by the Moroccan healthcare system. This literature review aims to examine and analyze the current state of telemedicine in Morocco. It seeks to highlight the challenges, initiatives, regulatory progress, and existing gaps in the landscape of telemedicine in Morocco.

## Introduction and background

The healthcare system in almost every country faces challenges in ensuring equitable access to quality healthcare services and in reducing or at least controlling the rising cost of healthcare services. Telemedicine can address both these needs by optimizing the use of existing human and material resources through telecommunications [1]. The application and use of computer technologies in healthcare is undergoing an evolutionary process. Advances in information technology, telecommunications, and networks have led to the emergence of a revolutionary new medical paradigm known as e-health [2].

According to the World Health Organization (WHO), e-health is the safe and cost-effective use of information and communication technologies (ICTs) to support health actions [1]. A branch of e-health, telemedicine is the use of new ICTs to make healthcare accessible to as many patients as possible, regardless of their location [3]. It connects distant services within the same country or across different countries. It enables communication between one or more healthcare professionals among themselves or with a patient. Among these professionals, a doctor is necessarily involved. The professionals provide care to the patient under the responsibility of their primary physician. It allows for diagnoses to be established, expert opinions to be obtained, treatment decisions to be made, services and care activities to be implemented, and patient monitoring to be conducted. It also facilitates the clinical training of healthcare professionals [4]. People seeking medical consultations on the one hand and those providing medical consultations on the other hand require a telemedicine infrastructure that relies on telecommunications tools. The speed and power of telecommunication lines and telemedicine equipment are major determinants of the scope of medical care [1].

Today, the gradual increase in the healthcare needs of the national population poses a major challenge to the Moroccan healthcare system, given the shortage of personnel in healthcare facilities and persistent difficulties in access in certain areas. In this context, and to meet these needs, the use of telemedicine emerges as an innovative and appropriate means to address this situation [5]. In Morocco, telemedicine is a part of a context marked by the need to improve access to quality healthcare for all citizens across the country, including in remote areas; the digitalization of the healthcare and social protection sector; a new development model in which telemedicine is one of the means to enhance healthcare delivery in terms of access and quality; and the overall guidelines for digital development in Morocco by 2025, developed by the Digital Development Agency [6,7].

This paper has been accepted as a poster at the 2023 European Federation for Medical Informatics (EFMI) and presented on October 26, 2023.

## Review

Territorial disparities in healthcare provision

The transformation of the healthcare sector aims, among other objectives, to mitigate social and spatial disparities in accessing essential healthcare [8]. This entails creating favorable conditions to enhance both the demand and supply of healthcare services. Roughly 270 rural municipalities experience critical healthcare isolation, signifying that they are located more than one hour away from a hospital facility. Out of these municipalities, 160 are classified as priority areas, encompassing around two million residents [9,10]. Since the early 2000s, Morocco has undertaken various projects and action plans directly or indirectly focused on reducing disparities, both overall and specifically in healthcare [5]. The Sustainable Development Goals, notably Goal 3 on health, present new challenges for Morocco. The country must uphold its commitment to enhance the health and well-being of all citizens, reduce inequalities, and ensure that no one is left behind [11]. The equity of healthcare access in Morocco, encompassing the distribution of healthcare resources across different regions, is highlighted in the framework law No. 34-09 on the healthcare system and the provision of healthcare services (July 21, 2011), promulgated by Dahir No. 1-11-83 on Rajab 29, 1432 (July 2, 2011) [12]. In 2015, the government introduced a program aimed at reducing territorial and social inequalities in rural areas, with the objective of connecting rural and mountain populations and improving their living conditions, including bolstering medical infrastructure [5,13].

Shortage of human resources

The healthcare system in Morocco is currently grappling with a noticeable resource shortage, particularly concerning human resources. The physician density stands at 0.9, and the nurse density is 0.84 per 1,000 inhabitants [14]. At present, Morocco has 28,892 physicians, resulting in an approximate ratio of 7.8 physicians per 10,000 inhabitants, although the WHO recommends a minimum of 23 physicians per 10,000 inhabitants [15]. The Casablanca-Rabat corridor attracts over half of physicians, underscoring significant disparities between urban and rural regions [10]. The deficit of human resources in the healthcare sector currently exceeds 97,000 healthcare professionals, encompassing 32,000 doctors and 65,000 nurses. The digitization of healthcare systems ushers in new possibilities for accessing medical services. Telemedicine, in particular, offers the potential to reshape the healthcare landscape and achieve territorial rebalancing in favor of areas with low medical density [6].

The regulatory framework governing the practice of telemedicine

The Ministry of Health and Social Protection recognizes the significant role of telemedicine as a catalyst to promote universal and improved access to healthcare, especially for individuals living in remote areas and areas affected by human resource shortages. Therefore, it has established a regulatory framework for the practice of telemedicine in Morocco. 

Decrees and Laws Regarding the Practice of Telemedicine

Morocco stands out as one of the few countries in Africa and the Arab world to have established a regulatory framework to oversee the practice of telemedicine [16]. The legal framework of telemedicine has been established by Law No. 131-13 related to the practice of medicine (Dahir No. 1-15-26 of Rabii 11, 1436 (February 19, 2015) promulgating Law No. 131-13 related to the practice of medicine (Articles 99 to 102)). Article 99 defines telemedical practice. It is specified that telemedicine is one of the forms of medical practice. It is explicitly stated that this practice does not deviate from the ethical rules of medical practice in force in Morocco. Article 100 defines the modalities of collaboration, through this new form of practice, with foreign doctors outside of the Moroccan territory. The objective is to "officially and legally" benefit from the expertise of foreign practitioners for advice or assistance. The article also specifies that professionals must be trained in telemedicine practice and have the technical skills required for the use of the corresponding device(s). Article 101 defines the ethics of the doctor-patient relationship within the context of telemedicine provision. The patient's consent, being necessary for telemedical acts, must be documented [17,18]. Decree No. 2-18 378 of Dhou al-Qi'dah 11, 1439 (July 25, 2018) concerning telemedicine has defined five telemedical acts and the conditions for the implementation and organization of telemedicine (in four chapters) [4]. Chapter 1 (articles 1-2) defines the acts of telemedicine (teleconsultation, teleexpertise, telesurveillance, teleassistance, and medical response). Chapter 2 (articles 3-9) defines the procedure for obtaining authorization for telemedicine and the appointment of the telemedicine commission that oversees the practice. Chapter 3 (articles 10-15) outlines the authorized institutions and professionals to practice telemedicine, the ethics of practice, and the responsibilities of practitioners regarding information governance (e.g., traceability, protection of medical confidentiality, and data confidentiality). Chapter 4 (articles 16-18) defines provisions to be taken by professionals wishing to practice telemedicine and the coverage of telemedical acts.

During the COVID-19 pandemic and in response to exceptional circumstances, the National Council of the Order of Physicians (CNOM) authorized the use of teleconsultation in patient monitoring, particularly for those with chronic illnesses, even in cases where there is no healthcare professional physically present with the patient, as required by the 2018 regulations. Consequently, the initial telemedicine decree from 2018 was modified, and several provisions were recently updated in a new decree that was adopted by the Council of Government on January 14, 2021, and published in the official journal on February 1, 2021 [17]. The key modifications pertain to the definition of teleconsultation, the composition of the telemedicine commission, and the elements of the authorization application file, which now require applicants to provide a copy of the prior authorization for the processing of personal data by the National Commission for the Control of Personal Data Protection (CNDP) [18].

Authorized Individuals and Entities for Telemedicine Practice

According to Moroccan laws, the following institutions are permitted to engage in telemedicine: public health services, university hospitals, nonprofit healthcare institutions, private healthcare institutions, and clinics or equivalent establishments. Physicians practicing in medical practices are also authorized to engage in telemedicine under the same conditions. However, a distinction must be made between those who can perform telemedical acts and those who can assist in them: all physicians registered with the Moroccan Medical Council or a foreign medical council (if required within the context of teleexpertise or teleassistance) can carry out telemedical acts, while all healthcare professionals recognized under Moroccan regulations can assist in telemedical acts. In both cases, professionals must be trained in telemedicine practice and possess the necessary technical skills for using corresponding devices. It should be noted that healthcare professionals participating in telemedicine acts can only perform acts that fall within their profession and for which they are authorized [17].

Stakeholders in the telemedicine ecosystem in Morocco

The telemedicine ecosystem in Morocco comprises a variety of stakeholders who play key roles, including (Table 1) patients, at the center of healthcare professionals' focus; public policy actors, including government ministries responsible for regulation, legislation, organization, and funding of telemedicine; regulatory bodies, encompassing public agencies and innovation actors responsible for establishing regulations to govern telemedicine; accelerators, such as the Moroccan Society of Telemedicine and the Moroccan Society of Telemedicine and e-Health, which act as mentors and contribute to the deployment of telemedicine initiatives in Morocco; funders, including government ministries, public agencies, and institutions, as well as funding organizations, primarily responsible for financing telemedicine projects and reimbursing medical expenses related to care provided through telemedicine; care providers, encompassing both public and private healthcare facilities responsible for delivering a range of telemedicine services to patients; and providers of telemedicine products and services, such as equipment and infrastructure providers, as well as telemedicine solution developers, among others (Report: Ministry of Health, Strategic Diagnosis of Telemedicine in Morocco, 2022).

**Table 1:** Stakeholders in the telemedicine ecosystem in Morocco

Stakeholder	Description
Patients	At the center of healthcare professionals' focus
Public policy actors	Including government ministries responsible for regulation, legislation, organization, and funding of telemedicine
Regulatory bodies	Encompassing public agencies and innovation actors responsible for establishing regulations to govern telemedicine
Accelerators	Such as the Moroccan Society of Telemedicine and the Moroccan Society of Telemedicine and e-Health, which act as mentors and contribute to the deployment of telemedicine initiatives in Morocco
Funders	Including government ministries, public agencies and institutions, as well as funding organizations, primarily responsible for financing telemedicine projects and reimbursing medical expenses related to care provided through telemedicine
Care providers	Encompassing both public and private healthcare facilities responsible for delivering a range of telemedicine services to patients
Providers of telemedicine products and services	Such as equipment and infrastructure providers, as well as telemedicine solution developers, etc.

Telemedicine initiatives in Morocco

Major Initiatives in the Public Sector

Several telemedicine initiatives have been implemented in Morocco, with a primary goal of targeting rural and remote areas. One of the major telemedicine initiatives in the public sector in Morocco is the national telemedicine initiative launched in October 2018. In partnership with the Moroccan Telemedicine Society (SMT), the Ministry of Health launched a comprehensive telemedicine program on October 22, 2018, aimed at providing healthcare services to isolated rural areas [5,13]. This initiative focuses on providing remote and real-time medical services performed by nurses at equipped dispensaries. Since March 2020, four of these sites have been operational, and eight others are currently being deployed. These dispensaries are in communication with assistant professors from Mohammed VI University of Health Sciences (UM6SS) at Sheikh Khalifa Hospital. The initiative also includes the provision of medication to residents in remote rural areas. By the year 2025, the National Telemedicine Initiative aims to cover 80% of medical deserts in Morocco (areas with access to a hospital by car within a minimum of two hours) and a target population of 1.5 million people [9,13].

Another telemedicine platform has been deployed in the Marrakech-Safi region since 2018 [19]. The program targets over four million individuals across eight provinces, with 60% of them residing in rural areas. The initiative involved equipping 28 sites distributed throughout the region with telemedicine technology to connect them to the Marrakech University Hospital. Teleconsultations have been conducted in numerous medical fields. Two connectivity strategies have been implemented for remote areas to overcome exclusion in underserved regions: satellite connectivity and the progressive deployment of 5G. Legally, the telemedicine legislation continually adapts and evolves, allowing for broader deployment and stronger data protection.

The telemedicine project titled "Mobile Ultrasound Patrol" aimed to enhance care for women in underdeveloped regions by identifying and treating fundamental causes of maternal mortality early on, highlighting the possibility of high-risk pregnancies [20]. The project was initiated by "QUALCOMM" in collaboration with partners in three rural regions of Morocco: Ribat El Khir, Oulmes, and Boulmane, where maternal mortality rates were high (72.6 deaths for every 100,000 live births). Using a portable ultrasound machine, a smartphone, or a tablet, gynecologists from Casablanca, Fès, and Meknès (as well as Paris) received and analyzed images to improve the management of high-risk pregnancies. The time and cost efficiencies gained, with no compromise in quality, improved the likelihood of early detection and treatment of the primary causes of maternal morbidity/mortality, potentially reducing the number of maternal and child deaths.

Another telemedicine initiative involves a mobile health pilot project for tuberculosis in the city of Salé (Rabat area) in collaboration with the Korea International Cooperation Agency [21]. This disease has a moderate incidence in Morocco. According to the WHO, its estimated incidence in 2021 was 35,000 cases, corresponding to an incidence rate of 94 per 100,000 inhabitants. This decline has been observed from a rate of 115 in 2000 to 94 in 2021. Despite this decline, the incidence of tuberculosis is not declining fast enough for the country to the goals outlined in the tuberculosis elimination strategy and Sustainable Development Goals by 2030 [22].The partnership agreement between the Moroccan League Fight Against Tuberculosis (LAT) and the South Korea Cooperation Agency was signed in March 2014 to deploy the "Mobile Health Tuberculosis" project. This innovative concept in tuberculosis treatment equips patients with a smart box that detects when patients discontinue their treatment. The Mobile Health Tuberculosis project offers a straightforward and cost-effective approach to closely monitoring patients and enhancing coordination among stakeholders.

Peritoneal Dialysis Initiative: The University Hospital of Fes has chosen to implement peritoneal dialysis alongside hemodialysis for the monitoring of patients with renal insufficiency [23]. Chronic kidney disease is a real public health problem in Morocco by its medical and socioeconomic consequences. It affects 2.9% of the population aged between 20 and 70 years old. Its principal causes are identified with diabetes (32.8%), hypertension (28.2%), and urinary lithiasis. Hypertension affects 16.7% of adults, diabetes affects 13.8%, and obesity affects 23.2% [24]. In the context of this telemedicine initiative, a device is provided to the patient at home, enabling doctors and nurses to remotely monitor the patient's data through data connectivity. The physician/nurse has access to indicators and can identify necessary actions.

The STROKOFES application: It assists neurologist practitioners outside the university hospital in diagnosis and decision-making, calculates medication dosages, and generates a PDF file containing the patient's clinical data, which can be sent to an expert neurologist at the university hospital for consultation. This allows them to benefit from their colleagues' expertise while remaining autonomous [25].

Several other initiatives were also launched, covering various fields, such as teleoncology, teleradiology, telecardiology, and teleechography [20]. However, most of them have never been scaled up to a large extent.

Tbib24 is an initiative led by the Ministry of Health, the National Council of Order of Physicians (CNOM), and a private partner to ensure the continuity of care during the confinement period. The platform brings together volunteer doctors from various specialties who provide medical advisory services to patients [26].

Major Initiatives in the Private Sector

Today, we cannot discuss genuine private deployments of telemedicine. No private medical entity or private doctor has yet (to our knowledge) received authorization for such endeavors. With the onset of the pandemic in Morocco and the implementation of lockdown measures, both patients and doctors found themselves in a situation of disconnection (sometimes fueled by patient apprehensions) from their doctor-patient relationship. In order to assist their patients in maintaining continuity of care or for other reasons, several practitioners turned to communication tools (sometimes dedicated platforms, but mostly social messaging tools) for teleconsultations. In this specific pandemic scenario, several technological platforms emerged that, according to the law, cannot be considered telemedicine providers. These platforms lack regulatory compliance, organizational alignment, and technical conformity. Some of them do not adhere to basic security and confidentiality requirements and operate outside the legal framework of telemedicine (e.g., Dabadoc.com, Med.ma, Tdwiza.com, and Avis-médical.ma) (Report: Ministry of Health, Strategic Diagnosis of Telemedicine in Morocco, 2022).

Barriers to the development of telemedicine in Morocco

The rules and conditions for reimbursement of telemedicine services by paying institutions are not defined: The decree states that the costs associated with telemedicine services should be covered, but it does not provide details on the reimbursement modalities. In addition, telemedicine services are not mentioned in the list of reimbursable procedures by social security institutions. The regulatory provisions of the telemedicine decree do not specify the concepts of pricing and billing: The regulatory provisions of the telemedicine decree do not detail the notion of fees and leave ambiguity regarding the applicable scales. The responsibility for telemedicine acts in the case of tele-expertise is solely attributed to the requesting physician, which may discourage practitioners in this regard. There is no online prescription sending tool to pharmacists for prescribing medications in the context of telemedicine. The digitized medical record is not widespread across all healthcare facilities. There is no national catalog of certified/approved platforms that can be used by healthcare professionals in the practice of telemedicine. The delay in digitizing patient pathways and the absence of national standards and norms for patient data hosting and protection are barriers to the development of telemedicine. In Morocco, the protection of personal data is not explicitly addressed in the decree related to telemedicine. Therefore, it is governed by Law No. 09-08 concerning the protection of individuals regarding the processing of personal data. Preserving personal data remains a major concern linked to the digitization of healthcare and the security of health data. Once digitized, health data naturally become vulnerable to various types of cyberattacks. Therefore, it is crucial for this patient data to be hosted in a way that ensures its security and confidentiality. The absence of well-defined hosting standards by authorities poses a significant challenge to the development of telemedicine in Morocco [4,19] (Report: Ministry of Health, Strategic Diagnosis of Telemedicine in Morocco, 2022).

Recommendations for the success of telemedicine in Morocco

To expedite the progress toward a future scenario where telemedicine activities are efficiently conducted in both public and private sectors, aligning with the country's endeavors to enhance healthcare services, here are some recommendations put forth in this regard (Table 2) [9] (Report: Ministry of Health, Strategic Diagnosis of Telemedicine in Morocco, 2022).

**Table 2:** Recommendations for the success of telemedicine in Morocco

Success factors	Recommendations
Regulatory and governance aspect	Enhance "strategic" governance under the Ministry of Health and Social Protection to monitor the progress of telemedicine projects across various domains (medical, technical, organizational, legal, economic, etc.). Establish "operational" regional bodies under the umbrella of the "strategic" body. Incorporate private medical practice into the healthcare organization framework. Describe the patient care pathway and the processes involved in each telemedicine procedure to share a common vision of telemedicine practices. Consider the alignment of supply/need/demand in telemedicine deployment. Include telemedicine as a mandatory component in regional medical plans.
Regulatory aspect	Update the list of regulatory classifications for telemedicine procedures. Define rules and conditions for the reimbursement of telemedicine procedures by paying institutions. Provide detailed guidelines for pricing and billing of telemedicine procedures. Clarify the responsibility for telemedicine acts in the case of tele-expertise (requesting physician versus required physician). Establish a patient consent process and ensure alternative care in case of refusal. Strengthen the data protection framework for telemedicine patient data, including the enactment of a specific law for patient data protection.
Operational aspect	Operationalize the process of obtaining authorization for telemedicine practice. Define a process for homologation and certification of telemedicine technical platforms. Involve national entities, such as the National Data Protection Commission (CNDP) and the Digital Development Agency (ADD), in the homologation process.
Human resources	Integrate a dedicated telemedicine curriculum into the healthcare training policy. Implement incentivizing tools for healthcare professionals who engage in telemedical practices.
Technical aspect	Transition toward a digital prescription model by implementing an online prescription sending tool and acceptance of treatment by pharmacists. Accelerate the widespread adoption of digital medical records as a prerequisite for broader telemedicine deployment. Establish standards and norms for healthcare data hosting and patient data protection. Envision a national catalog of certified/homologated platforms.

## Conclusions

Telemedicine in Morocco holds great promise for the country's healthcare system. While still in its early development, it is gaining significant attention from healthcare professionals and policymakers, leading to the establishment of various telemedicine initiatives at the national level. Today, technology provides the opportunity to benefit from telemedicine, which is becoming increasingly prevalent worldwide with the ubiquitous access to the Internet, allowing for a direct interaction with the treating physician or receiving necessary medical care remotely. Telemedicine, which is essentially a digitized continuation of healthcare services, should be based on clearly identified needs by public authorities to improve access to healthcare. The deployment of telemedicine should not prioritize technology at the expense of a sound approach to establishing a healthcare service offering. It is therefore time to pay more attention to the human resources of the healthcare system and provide appropriate and specialized training to support and adapt to rapid technological advancements in order to contribute to the improvement and popularization of healthcare delivery.
